# *Notes from the Field*: Increased Incidence of Fentanyl-Related Deaths Involving *Para*-fluorofentanyl or Metonitazene — Knox County, Tennessee, November 2020–August 2021

**DOI:** 10.15585/mmwr.mm7104a3

**Published:** 2022-01-28

**Authors:** Jordan Trecki, Roy R. Gerona, Ross Ellison, Chris Thomas, Darinka Mileusnic-Polchan

**Affiliations:** ^1^Drug and Chemical Evaluation Section, Diversion Control Division, Drug Enforcement Administration, Springfield, Virginia; ^2^Clinical Toxicology and Environmental Biomonitoring Laboratory, University of California, San Francisco, California; ^3^Knox County Regional Forensic Center, Knoxville, Tennessee.

Data from the National Center for Health Statistics indicate that drug overdose deaths in the United States have increased by 28.5% during 2020–2021, from 78,056 during a 12-month period ending in April 2020 to 100,306 during the same period in 2021 (*1*). Approximately 75% of drug overdose deaths during 2020 involved opioids. With illicitly manufactured fentanyl continuing to supplant the heroin market, and increasing illicit use of counterfeit pills containing either fentanyl, various fentanyl-related compounds, or other opioids, the risk for drug overdose deaths remains high (*2*). Recently, *para*-fluorofentanyl, a substance from research efforts in the 1960s and referred to as “China-white” before it was classified as a schedule I substance in 1986, has begun to reemerge on the illicit drug market.* It is found in heroin packets, counterfeit pills, and is reported in autopsy findings and supporting toxicology results.^†^ More recently, another class of compounds known as benzimidazole-opioids have begun appearing in cities across the country as adulterants in the heroin supply, adding a new threat to public health (*3*). Discovered through research for new opioid analgesics in the late 1950s, this class of opioid receptor agonists did not receive market authorization for therapeutic use. Based on law enforcement seizure data from the National Forensic Laboratory Information System (NFLIS)^§^ and medical examiner reports as described below, metonitazene, a benzimidazole-opioid, and *para*-fluorofentanyl (both in combination with fentanyl), are being encountered more often in the United States.

The Knox County Regional Forensic Center (KCRFC) in Knoxville, Tennessee is the medical examiner for Knox and Anderson counties, and also conducts autopsies when requested from 21 surrounding counties. KCRFC first identified *para*-fluorofentanyl in toxicology results of victims of drug overdoses in November 2020, and metonitazene, either alone or in combination with fentanyl and *para*-fluorofentanyl, in January 2021. During November 2020–August 2021, KCRFC reported 770 total unintentional drug overdose deaths. Among these deaths, 562 (73.0%) cases received postmortem positive test results for fentanyl (Table), including 192 fentanyl-positive cases reported in the absence of other substances (i.e., fentanyl only), 188 that involved fentanyl and methamphetamine, 48 that involved *para*-fluorofentanyl, and 26 that involved metonitazene.

**TABLE:** Demographic characteristics, circumstances, and co-occurring substances among overdose decedents with fentanyl, *para*-fluorofentanyl, or metonitazene detected in postmortem toxicology — Knoxville, Tennessee and surrounding counties,* November 2020–August 2021

Characteristic	No. (%)
**Total postmortem cases with fentanyl, *para*-fluorofentanyl, or metonitazene present, alone or in combination**	572^†^ (100)
**Sex**	
Male	388 (68.5)
Female	184 (31.5)
**Race/Ethnicity**	
White, non-Hispanic	515 (90.0)
Black, non-Hispanic	44 (7.7)
Hispanic or Latino	11 (2.0)
Other, non-Hispanic^§^	2 (0.3)
**Age group, yrs**	
<25	30 (5.2)
25–34	153 (26.7)
35–44	160 (28.0)
45–54	128 (22.4)
≥55	101 (17.7)
**Route of administration^¶^**	
Injection	75 (13.1)
Ingestion	52 (9.1)
Snorting	27 (4.7)
Smoking	0 (—)
Unknown	418 (73.1)
Occurrence of substances**	
Fentanyl, total	562 (98.3)
*Para*-fluorofentanyl, total	48 (8.4)
Metonitazene, total	26 (4.6)
Fentanyl only	192 (33.6)
Fentanyl and cocaine only	24 (4.2)
Fentanyl, cocaine and other opioids^††^	4 (0.7)
Fentanyl and methamphetamine only	188 (32.9)
Fentanyl, methamphetamine, and cocaine	12 (2.1)
Fentanyl, methamphetamine, and other opioids^§§^	32 (5.6)
Fentanyl and other stimulants^¶¶^	2 (0.3)
Fentanyl, methamphetamine, and other stimulants^¶¶^	6 (1.0)
Fentanyl and other opioids***	39 (6.8)
*Para*-fluorofentanyl only	3 (0.5)
Fentanyl and *para*-fluorofentanyl only	17 (3.0)
Fentanyl, *para*-fluorofentanyl, and metonitazene only	2 (0.3)
Fentanyl, *para*-fluorofentanyl and other opioids^†††^	4 (0.7)
Fentanyl, *para*-fluorofentanyl and other stimulants^§§§^	1 (0.2)
Fentanyl, *para*-fluorofentanyl and methamphetamine	15 (2.6)
Fentanyl, *para*-fluorofentanyl and cocaine	4 (0.7)
Fentanyl and metonitazene only	4 (0.7)
Fentanyl, *para*-fluorofentanyl and methamphetamine, and cocaine	1 (0.2)
Fentanyl, *para*-fluorofentanyl and methamphetamine, and metonitazene	1 (0.2)
Fentanyl, metonitazene, and cocaine	1 (0.2)
Fentanyl, metonitazene, and methamphetamine	12 (2.1)
Fentanyl, metonitazene, and other opioids^¶¶¶^	1 (0.2)
Metonitazene only	2 (0.3)
Metonitazene and methamphetamine	3 (0.5)
Fentanyl and other benzimidazole-opioids****	2 (0.3)
**Location**	
Knox County	304 (53.1)
Blount County	53 (9.3)
Anderson County	45 (7.9)
Sevier County	40 (7.0)
Monroe County	10 (1.7)
Other counties^††††^	120 (21.0)
**NFLIS-Drug, Tennessee reports /Total U.S. reports (%) (2015–2021)** ^§§§§^	
***Para*-fluorofentanyl**	
2015–2019	0/19 (—)
2020	19/133 (14.3)
2021	186/2,561 (7.3)
**Metonitazene**	
2015–2019	0/0 (—)
2020	46/109 (42.2)
2021	101/439 (23.0)

**Abbreviations**: MDA = 3,4-methylenedioxyamphetamine; MDMA = 3,4-methylenedioxymethamphetamine; NFLIS = National Forensic Laboratory Information System.

* The drug deaths listed in the table are not comprehensive of the actual drug related death totals for counties other than Knox and Anderson because medical examiners from these counties did not refer every drug overdose case to the Knox County Regional Forensic Center.

^†^ Includes eight cases in which *para*-fluorofentanyl or metonitazine were identified in the absence of fentanyl.

^§^ Asian , American Indian or Alaska Native, or Native Hawaiian or Other Pacific Islander.

^¶^ Based on drug paraphernalia present at the scene.

** Values will sum to >100% to account for various combinations.

^††^ Other opioids include 6-monoacetylmorphine, dihydrocodeine, hydrocodone, hydromorphone, methadone, mitragynine, morphine, oxycodone, and oxymorphone.

^§§^ Other opioids include morphine, oxycodone, and oxymorphone.

^¶¶^ Other stimulants included amphetamine, MDA, and MDMA.

*** Other opioids include acetyl fentanyl, buprenorphine, codeine, hydrocodone, hydromorphone, methadone, mitragynine, oxycodone, oxymorphone, and tramadol.

^†††^ Other opioids include 6-monoacetylmorphine, acetyl fentanyl, buprenorphine, dihydrocodeine, hydrocodone, methadone, morphine, oxycodone, oxymorphone, and tramadol.

^§§§^ Other stimulants include amphetamine.

^¶¶¶^ Other opioids include dihydrocodeine and hydrocodone.

**** Isotonitazene or protonitazene, other benzimidazole-opioids, were identified in two cases.

^††††^ Number of detections in other counties served by the Knox County Regional Forensic Center: Bradley = five; Campbell = 11; Cocke = 12; Fentress = two; Grainger = six; Hamblen = 10; Jefferson = 12; Loudon = 12; McMinn = nine; Meigs = one; Morgan = nine; Polk = two; Rhea = six; Roane = 17; Union = six.

^§§§§^ Data were queried on January 21, 2022; data reporting for 2021 is ongoing.

These findings demonstrate the contribution of both *para*-fluorofentanyl and the benzimidazole-opioid metonitazene to unintentional overdose deaths in eastern Tennessee. NFLIS data indicate a sharp increase in law enforcement encounters with *para*-fluorofentanyl and metonitazene from 2020 to 2021; reporting from late 2021 is in process. Although the percentage of law enforcement encounters with these substances in Tennessee decreased relative to the national total percentage within this time frame, the increase in encounters both within Tennessee and nationally reflect an increased distribution of *para*-fluorofentanyl and metonitazene throughout the United States. With *para*-fluorofentanyl and metonitazene each alone being capable of producing respiratory depression leading to death, the various combinations of these substances, in addition to possible other opioids including fentanyl-related compounds or adulterants included in each drug exhibit that could cause or exacerbate serious adverse effects, pose an even greater potential harm to the patient than that previously observed. Naloxone still serves as an effective drug to reverse opioid overdose; however, additional doses of naloxone might be required when stronger opioids like fentanyl, *para*-fluorofentanyl, metonitazene, or other benzimidazoles are involved or combined (*4*). 

Physicians, medical examiners, and toxicology laboratories should be aware of the increased presence of *para*-fluorofentanyl and the benzimidazole class of opioids^¶^ when treating patients in the emergency department or identifying these substances postmortem. Increasing public awareness regarding the fatal consequences after the ingestion of fentanyl, *para*-fluorofentanyl, metonitazene, and other opioids in addition to expanded naloxone availability should be prioritized to reduce opioid-related deaths both in Tennessee and throughout the United States.

## Footnotes

* https://www.deadiversion.usdoj.gov/schedules/orangebook/c_cs_alpha.pdf (Accessed January 21, 2022).

†
https://www.dea.gov/sites/default/files/2020-06/Synthetic%20Opioids-2020.pdf

§ The National Forensic Laboratory Information System (NFLIS) is an important resource in monitoring illicit drug trafficking, including the diversion of legally manufactured pharmaceuticals into illegal markets. NFLIS-Drug is a comprehensive information system that includes data from forensic laboratories that handle approximately 98% of an estimated 1.5 million distinct annual federal, state, and local drug analysis cases. NFLIS-Drug includes drug chemistry results from completed analyses only. https://www.nflis.deadiversion.usdoj.gov/

¶
https://www.deadiversion.usdoj.gov/drug_chem_info/benzimidazole-opioids.pdf

## References

[1] National Center for Health Statistics. Drug overdose deaths in the U.S. top 100,000 annually. Atlanta, GA: US Department of Health and Human Services, CDC; 2021. https://www.cdc.gov/nchs/pressroom/nchs_press_releases/2021/20210714.htm

[2] Trecki J. A perspective regarding the current state of the opioid epidemic. JAMA Netw Open 2019;2:e187104. 10.1001/jamanetworkopen.2018.7104 30657528

[3] Ujváry I, Christie R, Evans-Brown M, DARK classics in chemical neuroscience: etonitazene and related benzimidazoles. ACS Chem Neurosci 2021;12:1072–92. 10.1021/acschemneuro.1c00037 33760580

[4] Substance Abuse and Mental Health Services Administration. SAMHSA opioid overdose prevention toolkit. Rockville, MD: US Department of Health and Human Services; Substance Abuse and Mental Health Services Administration; 2018. https://store.samhsa.gov/sites/default/files/d7/priv/sma18-4742.pdf

